# Effect of Physical Activity on Adolescent Obesity Status over Time: A Latent Growth Modeling Approach

**DOI:** 10.3390/healthcare9081018

**Published:** 2021-08-07

**Authors:** Sophia Jihey Chung

**Affiliations:** Red Cross College of Nursing, Chung-Ang University, Seoul 06974, Korea; sjchung@cau.ac.kr

**Keywords:** physical activity, obesity, adolescents, latent growth modeling

## Abstract

This study aims to examine the longitudinal influence of physical activity (PA) on obesity among normal-weight adolescents in South Korea. A total of 1347 data samples from the Korean Children and Youth Panel Survey from 2011 to 2014 were used. Latent growth modeling was applied. PA was assessed by the hours spent on PA during physical education (PE) classes in the last week. Body mass index for age was considered to determine obesity status. PA during PE positively predicted the intercept of obesity status, but it was not associated with the slope of obesity status. Although a longitudinal effect of PA was not identified, PA could influence the initial status of obesity. Because obesity in early years could steadily influence future obesity status, early detection and intervention for adolescent obesity are necessary. Moreover, more studies examining the effect of PA during PE on obesity status among adolescents should be conducted to identify the association between PA during PE and obesity status over time.

## 1. Introduction

Globally, obesity has been known as one of the most serious health problems [1]. In particular, childhood obesity, including adolescent obesity, should be the focus of intervention, because it could persist into adulthood [2] and lead to diverse diseases such as hypertension, coronary heart disease, diabetes, and liver disease [3,4,5,6]. Obesity is also known to be associated with not only physical health problems but also psychosocial problems, including depression and bullying [7,8]. However, obesity could be prevented by modifying one’s behaviors [9]. Therefore, many countries, including South Korea, provide strategies to prevent adolescent obesity, although adolescent obesity still remains a serious health problem.

Increasing physical activity (PA) is one of the interventions for obesity. The effect of PA on obesity has been well recognized. Many studies have found that increasing PA and reducing sedentary behaviors are negatively associated with obesity across all age groups [10,11,12]. Although regular PA in adolescents is helpful to prevent obesity, the time spent on PA generally declines through adolescence [13]. Therefore, various programs have been developed in order to overcome or prevent adolescent obesity by increasing the levels of PA among adolescents. Given limited resources, many researchers have been trying to identify more effective approaches to encourage adolescents to engage in PA, such as family-centered or school-based strategies [10,12,14].

Engagement in PA during physical education (PE) classes is one of the strategies that researchers and policymakers have focused on, because adolescents are likely to spend more time in school than before. PE classes are usually provided in schools, where the adolescents’ level of PA can be easily monitored because of the regular contact and supportive facilities (gyms, playgrounds, etc.) that are available [12]. Previous studies with younger children have shown the positive effect of PA during PE on obesity [15,16]; however, fewer studies were found to examine the influence of the adolescents’ engagements in PA during PE on obesity. In addition, little is known about the effect of PA during PE on obesity over time, because most studies were cross-sectional. The longitudinal impact of PA on obesity should be explored to design more appropriate prevention programs targeting adolescents. Furthermore, by understanding the effects of PA on obesity over time, we could develop more effective school-based obesity prevention PA programs. Thus, the aim of this study was to examine the longitudinal effect of PA on obesity levels among adolescents in South Korea.

## 2. Materials and Methods

### 2.1. Study Participants

Data from the Korean Children and Youth Panel Survey (KCYPS), a nationally representative survey conducted by the National Youth Policy Institute (NYPI) [17] from 2011 to 2014, were used in this study. KCYPS was an annually tracked survey that ran from 2010 to 2016, and it features nationwide multistage cluster sampling [17]. At the beginning of the study in 2010, a total of 7071 students (2351 first graders, 2378 fourth graders, and 2342 seventh graders) were enrolled. In the final survey in 2016, the original sample retention rate was 80.0–85.5%.

The survey consisted of questions on individual developments including physical, intellectual, and psychosocial development and lifestyle, as well as environmental development related to family, friends, and community [17]. The survey contents differed slightly depending on the students’ grades, considering the participants’ ability to respond to the questions and their developmental stage.

The participants’ grade and wave were considered when the study sample was selected. In this study, students in the seventh grade were only considered when they were enrolled in the first wave of the survey in 2010. The data of waves 2–5 from 2011–2014 were used because some of the study variables were only assessed during these waves. Adolescents were asked to answer a self-reported questionnaire for each wave. The participants who did not completely answer the questions related to the study variables for each wave were excluded. In addition, after computing body mass index (BMI)-for-age based on their answers to the height and weight questions, adolescents who were classified as normal weight (from the 5th to the 85th percentiles) at the baseline of this study were included in this analysis. As a result, a total of 1347 students were included in this study. About half of them were male (48.6%), and most of the participants were 14 years old (87.7%).

### 2.2. Measurements

In this study, BMI-for-age percentiles were considered to determine participants’ obesity status. Considering the participants’ sex, age, and answers to the height and weight questions, a BMI-for-age percentile for each participant was calculated based on the Korea Disease Control and Prevention Agency growth charts [18].

For PA, the duration of PA during PE classes was assessed. The adolescents were asked to answer how many hours they engaged in PA during PE classes in the last week. Possible responses were 0 h, 1 h, 2 h, 3 h, and 4 h and over.

### 2.3. Data Analysis

Data were analyzed using SPSS version 26.0 and AMOS 26.0 (IBM Corp., Armonk, NY, USA). Descriptive statistics were used for obesity status and PA. Latent growth modeling (LGM) was applied to examine the effects of PA on obesity status over time. First, the unconditional models of the mean and variance of the variables were identified based on the model fit indices. After the confirmation of the significance of the slope variance, indicating that the changes of the variables could differ, the conditional model was tested. A chi-square statistic index, comparative fit index (CFI), Tucker and Lewis index (TLI), and root mean square error of approximation (RMSEA) were used to determine the adequate model for this analysis. CFI and TLI should be larger than 0.90 [19]. An RMSEA value of about 0.05 or less indicates a close fit of the model to the data, while one of about 0.08 or less suggests a reasonable error of approximation. If the RMSEA value is greater than 0.10, the model is considered inappropriate [20].

## 3. Results

### 3.1. Descriptive Statistics of PA and Obesity Status over Time

The means and standard deviations, skewness values, kurtosis values, and correlation coefficients of PA and obesity status at each time point are provided in Table 1. Obesity status appeared to increase, while PA during PE appeared to decrease across the assessment points. Based on skewness and kurtosis, all of the variables were assumed to be normally distributed. According to the correlational matrix, PA during PE at the last two assessment points was related with obesity status.

### 3.2. Unconditional Models of PA and Obesity Status

Table 2 provides the values for the unconditional models of PA and obesity status. For PA, the no-growth model showed the best model fit: χ^2^ (5) = 48.44, *p* < 0.001, CFI = 0.94, TLI = 0.92, and RMSEA = 0.08. The initial point (2.03, *p* < 0.001) was close to the mean of PA from Table 1 (2.03).

The linear model of obesity was better than the no-growth model: χ^2^ (8) = 46.47, *p* < 0.001, CFI = 0.99, TLI = 0.99, and RMSEA = 0.07. The initial point of obesity status was 37.48 (*p* < 0.001), and the slope was 4.71 (*p* = 0.002), implying that the BMI percentiles increased with age. As the variances of the initial status and slope of obesity status were statistically significant, adolescents’ obesity statuses differed from each other over time, and other variables, such as PA, could explain this phenomenon.

### 3.3. Conditional Model of Obesity Status

**Figure 1:**
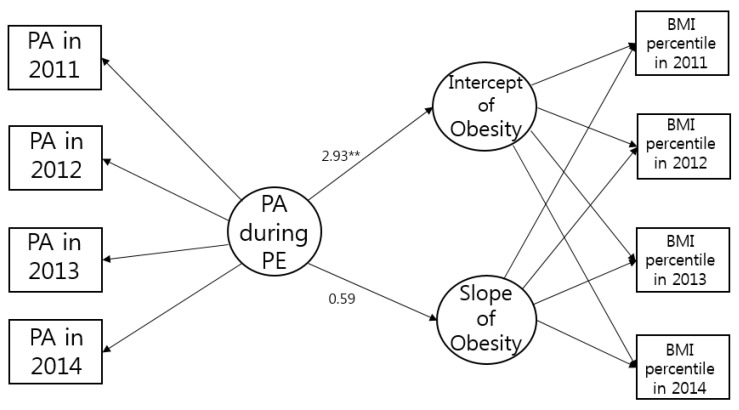
Final model of obesity status. ** *p* < 0.01.

The conditional model of obesity status over time also showed evidence for close model fit: χ^2^ (23) = 71.79, *p* < 0.001, CFI = 0.99, TLI = 0.99, and RMSEA = 0.04 (Figure 1). PA during PE positively predicted the intercept of obesity (β= 2.93, *p* = 0.009). That is, PA during PE could influence the initial obesity level among adolescents. However, it was not associated with the slope of obesity status (β= 0.59, *p* = 0.164), indicating the effect of PA during PE on obesity over time was not found.

## 4. Discussion

The aim of this study was to examine the effect of PA during PE on the obesity status of South Korean adolescents over time. Based on the final model proposed in this study, PA was significantly associated with initial obesity status. However, a longitudinal effect of PA was not identified.

Although no growth model was found to be the best model fit to explain the changes of PA, the duration of PA during PE declined from 2.03 in 2011 to 1.49 in 2014. The declining level of PA among adolescents could be found in other countries. According to a World Health Organization report in 2017, the levels of moderate-to-vigorous physical activity among European adolescents were low and declined with age, while the screen time, such as television viewing and computer use, increased over time [13]. Another previous study examining the trends in physical activity among adolescents from 146 countries found that most adolescents did not meet the physical activity guidelines, in addition to no significant changes among girls from 2001 to 2016 [21]. Because insufficient physical activity is a modifiable risk factor for diverse physical and psychosocial diseases [3,4,5,6,7,8], this issue should be more focused upon and intervened with. Because adolescents could feel guilty or pressured during PE [22], the strategies to encourage adolescents to spontaneously engage in PA during PE should be examined to promote adolescents’ PA. Providing sport clubs or other extracurricular activities in school [23,24] could be alternatives for increasing adolescents’ PA.

Based on the findings from this study, the obesity status of adolescents is likely to increase over time. Adolescents’ present or future obesity status could be determined by their previous status. This is consistent with the findings from other previous studies that obesity status during early childhood could persist into adolescence and even adulthood [2,25]. Although diverse influential factors are associated with obesity, obesity-related behaviors such as PA are established in childhood [26,27]. Once individuals establish those behaviors as habits, they might find it difficult to replace them with healthier ones and thus become overweight or obese. Therefore, early detection and intervention for potential overweight or obesity among adolescents is vital for the prevention of future obesity.

In this analysis, PA during PE could not explain the changes in obesity status over time among South Korean adolescents. Although PA has been known as a modifiable risk factor for obesity [28], the effect of PA during PE on obesity has been controversial. Among elementary school students in the United States, PE was found to lower BMI-z scores and the probability of obesity [15]. However, for adolescents, some researchers have concluded that the frequency of PE classes is associated with BMI improvement [29,30], while others have reported no effect of PA during PE [31]. In a study conducted in the United States, children participating in PE showed greater energy expenditure, which could be effective for controlling their obesity status if PE met the guidelines for PA [32]. However, in this study, the hours spent engaged in PA during PE did not satisfy the guidelines for adolescents’ recommended PA of at least 60 min of moderate-to-vigorous activity per day [33]. As a result, the effect of PA on changes in obesity could not be identified. The duration and types of PA during PE should be discussed for better school-based programs. More studies exploring the effects of PA during PE according to PA guidelines for adolescents are necessary. Based on further studies, more effective PE could be developed to prevent adolescent obesity. In addition, South Korean adolescents are more likely to be encouraged to pursue academic excellence and thus spend more time sitting to study, a kind of sedentary behavior, than those of other countries [34]. There could be other potentially more influential factors, such as the amount of time spent engaging in sedentary behaviors, affecting changes in obesity status.

This study has several limitations. First, because this is a secondary data analysis, not all of the related variables were considered, and only brief assessments of a range of variables were performed. For example, PA during PE was measured by asking adolescents how many hours of PA they engaged in during the last week. The types of PA during PE (moderate PA, vigorous PA, etc.) were not considered. Second, the participants were asked to answer self-reported questionnaires, and the answers might have been biased. For example, BMI for age to determine obesity status was computed based on adolescents’ answers. However, the exact heights and weights might not have been reported because the adolescents might not have remembered the exact values. PA during PE was also assessed by self-reported items, and there could be recall biases between the exact time engaged in PA and their answers. Moreover, underestimation of true weights and overestimation of true heights might have occurred. Lastly, this study analyzed Korean adolescents only, so cultural differences could have affected the results. More studies need to be conducted in other cultures to obtain more general information related to the influence of PA during PE on obesity.

## 5. Conclusions

In this study, the effect of PA during PE on obesity status over time was examined in 1347 normal-weight adolescents from KCYPS data from 2011 to 2014 using LGM. A longitudinal effect of PA during PE on adolescents’ obesity status was not found. However, an effect of PA during PE on adolescents’ initial obesity status was identified. Previous obesity status could have a role in determining later status, and thus, early detection and intervention are needed for the prevention of adolescent obesity. In addition, to determine the precise impact of PA during PE on obesity among adolescents, additional studies considering the satisfaction of the guidelines for PA and other potentially related factors are necessary.

## Figures and Tables

**Table 1:** Descriptive statistics and correlational matrix of PA and obesity status (*n* = 1470).

	1	2	3	4	5	6	7	8
1. 1st obesity								
2. 2nd obesity	0.76 ***							
3. 3rd obesity	0.69 ***	0.76 ***						
4. 4th obesity	0.65 ***	0.74 ***	0.82 ***					
5. 1st PA	0.02	0.03	0.04	0.03				
6. 2nd PA	0.00	0.03	0.03	0.04 *	0.38 ***			
7. 3rd PA	0.08 **	0.10 **	0.09 ***	0.08 ***	0.28 ***	0.31 ***		
8. 4th PA	0.06 *	0.08 *	0.08 **	0.08 ***	0.24 ***	0.32 ***	0.38 ***	
Mean	38.32	37.55	41.06	42.38	2.03	1.95	1.60	1.49
Standard deviation	23.07	24.46	26.29	27.28	1.23	1.35	1.12	1.15
Skewness	0.36	0.49	0.40	0.39	−0.07	0.13	0.31	0.47
Kurtosis	−1.08	−0.79	−0.95	−0.96	−1.10	−1.18	10.51	−0.41

* *p* < 0.05, ** *p* < 0.01, *** *p* < 0.001, PA: physical activity.

**Table 2:** Model fitness index.

Variables	Model	χ^2^ (df)	CFI	TLI	RMSEA	Intercept		Slope	
						Mean	Variance	Mean	Variance
PA	Non	48.44(5) ***	0.94	0.92	0.08	2.03 ***	0.45 ***		
	Linear	100.11(8) ***	0.88	0.91	0.09	2.06 ***	0.68 ***	−0.59 ***	0.36 ***
Obesity Status	Non	279.87(5) ***	0.93	0.92	0.20	38.32 ***	471.24 ***		
	Linear	46.47(8) ***	0.99	0.99	0.07	37.48 ***	423.95 ***	4.71**	228.05 ***

** *p* < 0.01, *** *p* < 0.001, CFI: comparative fit index; TLI: Tucker–Lewis index; RMSEA: root mean square error of approximation.

## Data Availability

The Korea Youth Panel Survey data are publicly available from the National Youth Policy Institute at www.nypi.re.kr (accessed on 8 March 2021).
